# High-throughput mutational analysis of *TOR1A *in primary dystonia

**DOI:** 10.1186/1471-2350-10-24

**Published:** 2009-03-11

**Authors:** Jianfeng Xiao, Robert W Bastian, Joel S Perlmutter, Brad A Racette, Samer D Tabbal, Morvarid Karimi, Randal C Paniello, Andrew Blitzer, Sat Dev Batish, Zbigniew K Wszolek, Ryan J Uitti, Peter Hedera, David K Simon, Daniel Tarsy, Daniel D Truong, Karen P Frei, Ronald F Pfeiffer, Suzhen Gong, Yu Zhao, Mark S LeDoux

**Affiliations:** 1 Departments of Neurology and Anatomy & Neurobiology, University of Tennessee Health Science Center, Memphis, TN, USA; 2 Bastian Voice Institute, Downers Grove, IL, USA; 3 Department of Neurology, Washington University School of Medicine, St. Louis, MO, USA; 4 New York Center for Voice and Swallowing Disorders, New York, NY, USA; 5 Athena Diagnostics, Inc., Worcester, MA, USA; 6 Department of Neurology, Mayo Clinic, Jacksonville, FL, USA; 7 Department of Neurology, Vanderbilt University, Nashville, TN, USA; 8 Department of Neurology, Beth Israel Deaconess Medical Center and Harvard Medical School, Boston, MA, USA; 9 Parkinson's & Movement Disorder Institute, Fountain Valley, CA, USA

## Abstract

**Background:**

Although the c.904_906delGAG mutation in Exon 5 of *TOR1A *typically manifests as early-onset generalized dystonia, DYT1 dystonia is genetically and clinically heterogeneous. Recently, another Exon 5 mutation (c.863G>A) has been associated with early-onset generalized dystonia and some ΔGAG mutation carriers present with late-onset focal dystonia. The aim of this study was to identify *TOR1A *Exon 5 mutations in a large cohort of subjects with mainly non-generalized primary dystonia.

**Methods:**

High resolution melting (HRM) was used to examine the entire *TOR1A *Exon 5 coding sequence in 1014 subjects with primary dystonia (422 spasmodic dysphonia, 285 cervical dystonia, 67 blepharospasm, 41 writer's cramp, 16 oromandibular dystonia, 38 other primary focal dystonia, 112 segmental dystonia, 16 multifocal dystonia, and 17 generalized dystonia) and 250 controls (150 neurologically normal and 100 with other movement disorders). Diagnostic sensitivity and specificity were evaluated in an additional 8 subjects with known ΔGAG DYT1 dystonia and 88 subjects with ΔGAG-negative dystonia.

**Results:**

HRM of *TOR1A *Exon 5 showed high (100%) diagnostic sensitivity and specificity. HRM was rapid and economical. HRM reliably differentiated the *TOR1A *ΔGAG and c.863G>A mutations. Melting curves were normal in 250/250 controls and 1012/1014 subjects with primary dystonia. The two subjects with shifted melting curves were found to harbor the classic ΔGAG deletion: 1) a non-Jewish Caucasian female with childhood-onset multifocal dystonia and 2) an Ashkenazi Jewish female with adolescent-onset spasmodic dysphonia.

**Conclusion:**

First, HRM is an inexpensive, diagnostically sensitive and specific, high-throughput method for mutation discovery. Second, Exon 5 mutations in *TOR1A *are rarely associated with non-generalized primary dystonia.

## Background

Dystonia is a syndrome of sustained muscle contractions, frequently causing twisting and repetitive movements, or abnormal postures [1]. Dystonia can be classified by etiology (primary or secondary), age of onset (childhood-onset [0–12 yrs], adolescent-onset [13–20 yrs], or late-onset [>20 yrs]), and anatomical distribution (focal, segmental, multifocal, hemidystonia, or generalized) [1-4]. Primary generalized dystonias usually begin in childhood or adolescence, whereas focal dystonias typically present during adult life. Primary dystonia includes syndromes in which dystonia is the sole phenotypic manifestation, with the exception that tremor may be present as well.

In most neurology subspecialty practices, the vast majority of patients with dystonia are adults with primary focal or segmental disease. Genetic factors likely play a major role in late-onset primary dystonia since 8–27% of patients with primary late-onset dystonia have one or more family members affected with dystonia [5-9] and several of the primary dystonias inherited in Mendelian fashion (DYT1, DYT5, DYT6, DYT11, and DYT12) begin focally, show incomplete penetrance and exhibit variable anatomical expressivity [10-12]. These facts suggest that sporadic late-onset dystonia, much like Parkinson's disease, is a complex disorder with contributions from multiple genes and environmental factors. Candidate gene studies have been successful in several late-onset neurological disorders, particularly Parkinson's disease, and may be fruitful in primary dystonia. Late-onset sporadic dystonia could be associated with a number of distinct mutations of low penetrance.

The ΔGAG (c.904_906delGAG) mutation in Exon 5 of *TOR1A *is characteristically associated with early-onset generalized dystonia [13]. Although infrequent, the DYT1 ΔGAG mutation has also been associated with late-onset focal, segmental, and multifocal dystonia [14-19]. Another mutation in Exon 5 of *TOR1A *(c.863G>A) has been described in a female patient with severe childhood-onset generalized dystonia [20]. The G>A transition in Exon 5 results in exchange of an arginine for glutamine. In contrast to the DYT1 ΔGAG mutation, analysis of late-onset dystonia cases for the c.863G>A mutation has not been described to date. Two additional mutations have been described in Exon 5, the terminal exon of *TOR1A*. Leung and colleagues [21] reported a patient with early-onset dystonia and myoclonus who harbored an 18-bp deletion in Exon 5 which should eliminate 6 amino acids near the carboxy terminus of torsinA (Phe323_Tyr328del), including a putative phosphorylation site. The causality of the 18-bp deletion is unclear since the same subject was subsequently found to have an *SGCE *mutation [22]. As described in another study, a novel out-of-frame 4-bp deletion (c.934_937delAGAG) found in a putatively healthy blood donor should result in an alteration of amino acids starting at position 312 with a premature stop at position 325 (E312/Stop325) in the carboxy terminus of torsinA [18].

To help address the role of the *TOR1A *gene in non-generalized primary dystonia, we used high resolution melting (HRM) to examine the entire *TOR1A *Exon 5 coding sequence in a large cohort of patients with non-generalized dystonia. HRM has been shown to be a fast and accurate, closed-tube, post-PCR mutation scanning technique that monitors the progressive change in fluorescence caused by the release of an intercalating DNA dye from DNA duplexes as they are denatured by minor increases in temperature [23]. Our results indicate that mutations in Exon 5 of *TOR1A *are rare in non-generalized primary dystonia.

## Methods

### Subjects

All human studies were performed in accordance with institutional review board guidelines and all subjects gave informed consent. Subjects with dystonia and neurologically-normal controls were acquired from outpatient clinics at participating institutions (University of Tennessee Health Science Center, Bastian Voice Institute, Washington University School of Medicine in St. Louis, New York Center for Voice and Swallowing Disorders, Mayo Clinic Jacksonville, Beth Israel Deaconess Medical Center, Vanderbilt University and the Parkinson's & Movement Disorder Institute) and support group meetings of the National Spasmodic Dysphonia Association (NSDA), National Spasmodic Torticollis Association (NSTA), Benign Essential Blepharospasm Research Foundation (BEBRF), Dystonia/Spasmodic Torticollis, and Dystonia Medical Research Foundation (DMRF). All subjects with dystonia acquired at support group meetings were examined by M.S.L. Subjects with Parkinson's disease and other movement disorders were acquired from the clinics of M.S.L. and R.F.P. Clinical diagnoses and classifications were made by means of history and examination by one or more neurologists and/or neurolaryngologist at each institution. Neurologically-normal control subjects were defined as individuals with no personal or first-degree family history of neurological disease. In addition, all control subjects acquired at the University of Tennessee Health Science Center and support group meetings were examined by M.S.L. or R.F.P. Dystonia was classified in accordance with established schemes [1,3,24]. Subjects with established diagnoses of DYT1 dystonia were not recruited into our study.

Clinical diagnoses for 1264 subjects interrogated with HRM appear in Table 1. Demographic information and dystonia distribution was not available for 96 DNA samples received from Athena Diagnostics, Inc. (Worcester, MA). The panel from Athena Diagnostics included 8 samples with confirmed DYT1 ΔGAG deletions and 88 ΔGAG-negative samples associated with a clinical diagnosis of "dystonia."

**Table 1:** Clinical diagnoses and demographics

**Clinical diagnosis**	**Number (age of onset)^a^**	**Family history^b^**	**Gender**		**Race/Ethnicity**			***TOR1A *exon 5 mutations**
				
			**Male**	**Female**	**Non-Jewish** **Caucasian**	**Jewish**	**Other**	
Spasmodic dysphonia	422 (45.0 ± 15.7, 7–84)	7.3%	91	331	368	2	52	1 ΔGAG

Cervical dystonia	285 (43.7 ± 13.8, 4 – 76)	8.4%	71	214	268	1	16	0

Blepharospasm	67 (54.4 ± 10.1, 20–73)	10.4%	20	47	61	2	4	0

Writer's cramp	41 (35.3 ± 14.4, 7–60)	7.3%	17	24	35	0	6	0

Oromandibular dystonia	16 (48.9 ± 15.4, 20–70)	6.3%	3	13	13	1	2	0

Other primary focal dystonia	38 (37.8 ± 18.2, 10–74)	5.3%	14	24	34	0	4	0

Segmental dystonia	112 (48.7 ± 12.8, 14–74)	13.4%	39	73	101	0	11	0

Multifocal dystonia	16 (30.0 ± 15.9, 7–67)	25.0%	3	13	15	0	1	1 ΔGAG

Generalized dystonia	17 (18.2 ± 15.3, 1–57)	0.0%	7	10	17	0	0	0

**Dystonia totals**	**1014** **(44.3 ± 15.6, 1–84)**	**8.6%**	**265**	**749**	**912**	**6**	**96**	**2 ΔGAG**

Parkinson's disease	42 (63.0 ± 12.5, 28–82)	NA	19	23	39	0	3	0

Restless legs syndrome	31 (40.6 ± 18.3, 8–66)	NA	12	19	29	0	2	0

Essential tremor	14 (50.9 ± 15.4, 30–70)	NA	9	5	13	0	1	0

Other movement disorders	13 (49.8 ± 17.3, 27–71)	NA	9	4	12	0	1	0

Neurologically-normal controls	150 (56.5 ± 14.5, 23–83)^c^	NA	79	71	135	0	15	0

ΔGAG-negative dystonia	88 (NA)	NA	NA	NA	NA	NA	NA	0

ΔGAG DYT1 dystonia	8 (NA)	NA	NA	NA	NA	NA	NA	8 ΔGAG

**Grand totals**	**1360**							10 ΔGAG

^a^Mean +/- standard error, range (yrs).

^b^First- or second-degree relative with dystonia.

^c^Age at study enrollment.

NA = not available or applicable.

### DNA

DNA was extracted from peripheral blood leucocytes using Roche's DNA Isolation Kit for Mammalian Blood (Roche Applied Science, Indianapolis, IN). DNA quantity and quality were analyzed with a NanoDrop ND-100 spectrophotometer (NanoDrop Technologies LLC, Wilmington, DE), Quant-iT™ PicoGreen^® ^dsDNA Assay Kit (Invitrogen Inc. Carlsbad, CA) and agarose gel electrophoresis. High-quality DNA samples were diluted with PCR water to a concentration of 10 ng/μl. Poor quality samples were rescued by whole genome amplification with the REPLI-g^® ^Mini Kit from Qiagen (Valencia, CA). Samples that could not be rescued were not used for HRM and do not appear in Table 1.

### HRM

Using Primer3 (frodo.wi.mit.edu), a pair of PCR primers was designed to cover the entire *TOR1A *Exon 5 coding sequence (forward: cagcaccttgtttcttcttcc, reverse: ccaactccaggcagtgactc). Another forward primer was synthesized for site-directed mutagenesis of Exon 5 to generate the c.863G>A mutation (aacagcaccttgtttcttcttcccaggtggcttctggcacagcagcttaattgaccggaacctcattgattattttgttcccttcctccccctggaatacaaacacctaaaaatgtgtatccgagtggaaatgcagtcccAaggctatga). To evaluate HRM results with amplicons of different sizes, a pair of previously-published primers was used to amplify the DYT1 ΔGAG region of Exon 5 [13].

**Figure 1:**
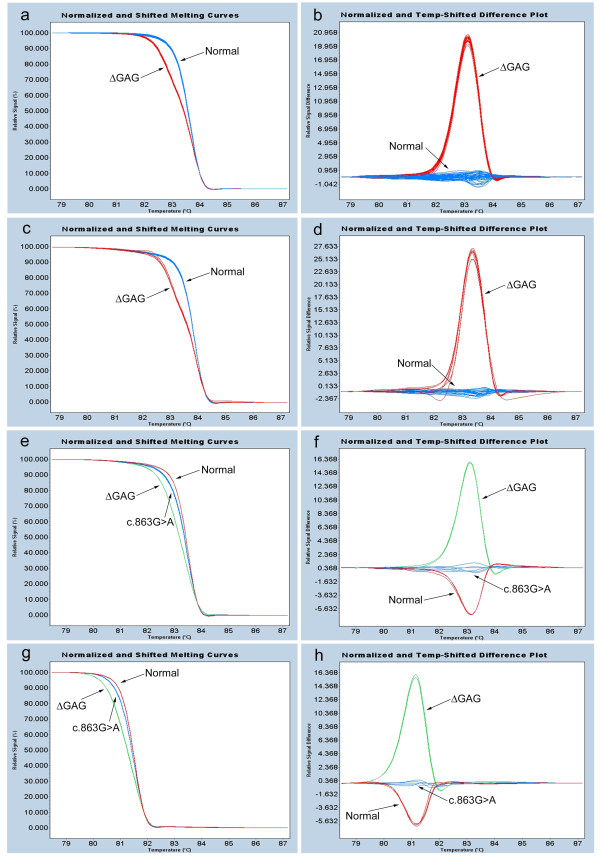
**HRM results of *TOR1A *Exon 5 coding region**. Normalized and temperature-shifted melting curves (a) and difference plots (b) for 8 ΔGAG-positive (red) and 88 ΔGAG-negative (blue) samples. Normalized and temperature-shifted melting curves (c) and difference plots (d) for one ΔGAG-positive (red) and five ΔGAG-negative (blue) samples with different concentrations of DNA template (0.1 ng/μl, 0.25 ng/μl, 0.5 ng/μl, 1 ng/μl, and 2.5 ng/μl; each in duplicate). Normalized and temperature-shifted melting curves (e) and difference plots (f) for ΔGAG-positive (green; one sample, in duplicate), ΔGAG-negative (red; three samples, each in duplicate) and c.863G>A mutation (blue; 1:1 mixtures of template DNA from normals with c.863G>A amplicons) 314 bp amplicons. Normalized and temperature-shifted melting curves (g) and difference plots (h) for ΔGAG-positive (green; one sample, in duplicate), ΔGAG-negative (red; three samples, each in duplicate) and c.863G>A mutation (blue; 1:1 mixtures of template DNA from normals with c.863G>A amplicons) 205 bp amplicons.

The LightCycler^® ^480 Real-Time PCR system and High Resolution Master Mix from Roche Applied Science (Indianapolis, IN, USA) were used for HRM. The master mix contains the LightCycler 480 ResoLight Dye. The High Resolution Melting Master Mix is a ready-to-use hot-start mix designed for PCR amplification followed by HRM curve analysis for detection of sequence variants. ResoLight, a novel saturating DNA dye, was specifically designed for detection of sequence variations by differences in melting curves. ResoLight can be used at high concentrations without inhibiting PCR and its homogeneous staining of target sequences results in sharp melting signals. As the temperature of the solution is increased during HRM, the specific sequence of the amplicon (primarily GC content and length) determines the melting behavior. When the fluorescence signal is plotted against temperature, fluorescence intensity decreases as double-stranded DNA (dsDNA) becomes single stranded and ResoLight is released. Since melting point (T_M_) differences are often too small for reliable differentiation of amplicons, separation is achieved by signal normalization and temperature shifting (Fig 1a, c, e, and 1g). For normalization, the pre- and post-melt signals are set to uniform relative values. For temperature shifting, the temperature axes of the normalized melting curves are shifted to the point where the entirety of dsDNA is denatured. The LightCycler 480 Gene Scanning Software detects differences in melting curves and allocates samples to groups of the same sequence. Finally, difference plots are created by subtracting melting curves from a reference curve (Fig 1b, d, f, and 1h).

The HRM Master Mix contains FastStart Taq DNA polymerase, reaction buffer, a dNTP mix and ResoLight. HRM was optimized to detect and differentiate the ΔGAG and c.863G>A mutations by adjusting reaction conditions and the concentrations of template DNA, primers and MgCl_2_. To emulate the heterozygous state, a 1:1 mixture of normal DNA with c.863G>A amplicons was utilized for these reactions. Then, to define sensitivity and sensitivity, HRM was performed with the collection of 96 samples from Athena Diagnostics that included 8 samples with confirmed DYT1 ΔGAG deletions and 88 additional samples with a clinical diagnosis of "dystonia." Optimized reactions were performed in 96-well plates (Roche Catalog #04729692001) using 10–20 ng of template DNA, 1× HRM Master Mix, 2.5 mM MgCl_2 _and 200 nM of each primer in a 20-μl reaction volume. PCR cycling and HRM conditions were carried out as follows: 95°C for 10 min; 45 cycles at 95°C for 10 s, 60°C for 15 s, and 72°C for 15 s; 95°C for 1 min, 40°C for 1 min, and the final HRM temperature ramp from 70°C to 95°C rising at 0.1°C/s. All subject samples and negative controls were run in duplicate using 96-well plates.

With LightCycler 480 Gene Scanning Software, melting curves and difference plots were analyzed by three investigators (S.G., Y.Z., and M.S.L.) blinded to phenotype. All samples were unambiguously assigned to one of three genotypes (wild-type or normal, ΔGAG deletion, or c.863G>A mutation) by Gene Scanning Software. DNA from wells with abnormal melting curves was purified with the Qiagen QIAquick PCR Purification Kit. The ΔGAG deletion and c.863G>A mutations were confirmed by direct sequencing of purified PCR products in the forward and reverse directions using an ABI Prism 377 DNA Sequencer (Applied Biosystems, Foster City, CA, USA).

## Results

### HRM

As seen in Fig 1a and 1b, all 8 ΔGAG-positive samples were clearly differentiated from the 88 ΔGAG-negative samples; amplicons containing the ΔGAG mutation melted/denatured prior to the ΔGAG-negative samples. The ΔGAG-positive samples clustered together on the melting curves and difference plots with minimal sample-to-sample variation. Furthermore, segregation of ΔGAG-positive and ΔGAG-negative samples was maintained despite 25-fold variation in the concentrations of DNA templates from 0.1 ng/μl to 2.5 ng/μl (Fig 1c and 1d). No false positive or false negative samples were detected within this broad concentration range.

HRM robustly distinguished samples with either ΔGAG deletions or c.863G>A mutations from normals (Fig 1e–1h). Diagnostic sensitivity and diagnostic specificity for ΔGAG deletion and c.863G>A mutation were 100% with both primer pairs (Fig 1e–1h). As expected, the larger *TOR1A *Exon 5 amplicon (314 bp, Fig 1e and 1f) had a higher melting temperature (T_M _= 83.2°C) than the smaller amplicon (205 bp, T_M _= 81.2°C) which was generated with previously published primers [13].

LightCycler^® ^480 HRM Master Mix (Catalog #04909631001) was purchased for $400  and contained reagents for 500 reactions (20 μl reaction volume, $0.80/reaction). LightCycler^® ^480 Multiwell Plates 96, white with sealing foils (Catalog # 04729692001), must be used for HRM and were purchased for $360/50 plates ($7.20/plate). Excluding the costs of pipette tips and primers, 96 reactions were completed for $84.00. On average, each plate was setup, run, and analyzed in 3 hrs.

### *TOR1A* Exon 5 Mutations in Non-Generalized Primary Dystonia

Two subjects (#1 and #2) with non-generalized primary dystonia were found to harbor the classic DYT1 ΔGAG deletion in Exon 5 of *TOR1A*. No *TOR1A *Exon 5 mutations or variants were identified in the remaining 1012 subjects with dystonia or 250 controls. Subject #1 with the classic DYT1 ΔGAG mutation exhibited multifocal dystonia. She was a 48-yr-old non-Jewish Caucasian female with cervical dystonia, spasmodic dysphonia (adductor subtype), and writer's cramp. Cervical dystonia became manifest at 12 yrs of age whereas laryngeal involvement and writer's cramp became apparent at 39 and 45 yrs of age, respectively. She reported consistent benefit from injections of botulinum toxin type A for treatment of her laryngeal and cervical dystonia. Her mother had never been diagnosed with "dystonia" but reportedly exhibited action-induced "leg tremors." A maternal aunt (her mother's monozygotic twin) suffered from an undiagnosed voice disorder prior to her death. Subject #1 had two siblings, both brothers. By report, one brother was neurologically normal. The second brother developed generalized dystonia at 9 years of age and died at 16 years of age with respiratory complications.

Subject #2, also with the classic DYT1 ΔGAG mutation, was a 63-yr-old woman of Ashkenazi Jewish descent. She had spasmodic dysphonia (adductor subtype) that became manifest at 19 years of age. Injections of botulinum toxin type A achieved consistently excellent results over a period of 12 yrs. Subject #2 also exhibited a bilateral upper extremity action tremor (<2 cm excursions). She had one child who was neurologically normal. Her mother and maternal grandmother had received diagnoses of "essential tremor." None of her family members were available for neurological examination.

## Discussion

In 2000, Bressman and colleagues published diagnostic testing guidelines for the DYT1 GAG deletion [25] and recommended DYT1 ΔGAG testing in conjunction with genetic counseling for subjects displaying primary dystonia with onset prior to 26 yrs of age. Testing may be warranted in subjects with onset after age 26 if they have an affected relative with early-onset primary dystonia [25]. Our results are entirely consistent with those guidelines. Although *de novo *ΔGAG mutations of *TOR1A *Exon 5 have been described [26,27], our findings indicate that they must be extraordinarily rare in non-generalized primary dystonia in the United States. Furthermore, we have shown that other *TOR1*A Exon 5 variants, including the recently described c.863G>A missense mutation, must also be rare in subjects with non-generalized primary dystonia.

As outlined in Table 2, the classic DYT1 ΔGAG mutation is uncommon in non-generalized primary dystonia and quite rare in late-onset primary dystonia. The typical DYT1 phenotype is characterized by early-onset in a limb, most commonly a leg, with spread to other limbs and the trunk over several years. Writer's cramp or more extensive upper limb dystonia without task-specificity is the most common presentation of adolescent- or late-onset primary dystonia due to the DYT1 ΔGAG mutation and most of these subjects will have a positive family history of dystonia [19]. However, DYT1 dystonia is phenotypically heterogeneous with oftentimes striking intrafamilial and interfamilial variability [28-31]. Indeed, our subject #1 had childhood-onset cervical dystonia with much later development of laryngeal dystonia and writer's cramp, whereas subject #2 manifested dystonia with isolated laryngeal involvement for over 40 yrs. Other remarkable phenotypes described in the literature include onset of focal dystonia at 64 yrs, status dystonicus, and late-onset dystonia precipitated by exposure to a neuroleptic [29,30].

**Table 2:** DYT1 mutations in non-generalized primary dystonia

**Reference**	**Number of subjects with non-generalized primary dystonia**	**Number with ΔGAG mutations**	**Phenotypic description of ΔGAG-positive subjects**
Valente et al. (1998) [15]	108 (Europe) S = 48, F = 60	3 (2.8%)	#1: S(arms), early-onset, FH+, Jewish #2: F(arm), early-onset, FH-, Jewish #3: F(arm), early-onset, FH+

Klein et al. (1999) [37]	300 (United States, Germany, Italy; late-onset & FH+) S = 7, F = 293	3 (1.0%)	#1: S(both legs & one arm), late-onset, FH+ #2: F(cranial), late-onset, FH+ #3: F(NA), late-onset, FH+, Jewish

Brassat et al. (2000) [39]	90 (France, FH-) S = 21, F = 69	1(1.1%)	#1: S(leg & trunk), early-onset, FH-

Kamm et al. (2000) [40]	44 (Germany) F = 44 (writer's cramp)	0 (0.0%)	NA

Friedman et al. (2000) [41]	18 (United States) F = 18 (musicians)	0 (0.0%)	NA

Major et al. (2001) [42]	38 (Serbia) M = 1, S = 22, F = 15	2 (5.3%)	#1: S(both arms), early-onset, FH- #2: M(right arm & left leg), early-onset, FH-

Matsumoto et al. (2001) [43]	173 (Japan) M = 4, S = 29, F = 144,	3 (1.7%)	#1: F(arm), early-onset, FH+ #2: F(arm), early-onset, FH+ #3: F(leg), late-onset, FH+

Maniak et al. (2003) [8]	130 (Germany) S = 10, F = 120	0 (0.0%)	NA

Grundmann et al. (2003)[17]	244 (Germany) M = 11, S = 46, F = 187	4 (1.6%)	#1: M(both legs & right arm), early-onset, FH- #2: M(both arms & cranial), late-onset, FH+ #3: S(both arms), early-onset, FH+ #4: S(both arms), early-onset, FH-

Kabakci et al. (2004) [18]	126 (Germany) F + S = 126	0 (0%)	NA

Im et al. (2004) [44]	155 (Korea) S = 21, F = 134	2 (1.3%)	#1: S(arm & shoulder), early-onset, FH+ #2: S(arm & neck), early-onset, FH-

Dhaenens et al. (2005) [45]	150 (France) F = 150	1 (0.7%)	#1: F(arm), onset?, FH+

Lin et al. (2006) [46]	186 (Taiwan) M = 3, S = 47, F = 136	2 (1.1%)	#1: F(leg), early-onset, FH+ #2: S(neck & trunk), late-onset, FH+

Jamora et al. (2006) [47]	54 (Singapore) M = 1, S = 11, F = 41	0 (0.0%)	NA

Naiya et al. (2006) [48]	150 (India) F = 138, S = 12	0 (0%)	NA

Gajos et al. (2007) [31]	48 (Poland) G + S + F = 48	F = 2 (NA)	#1: F(arm), late-onset, FH+ #2: F(arm), early-onset, FH+

G, generalized dystonia; M, multifocal dystonia; S, segmental dystonia; F, focal dystonia; FH, family history of dystonia; and NA, not applicable.

In comparison with several of the studies listed in Table 2, the percentage of ΔGAG mutations was relatively low in our cohort of subjects with non-generalized dystonia. Approximately, 90% of our patients with primary dystonia were non-Jewish Caucasians born in the United States; only 6 Jewish subjects were screened for *TOR1A *Exon 5 mutations. Since the carrier frequency of the classic DYT1 ΔGAG mutation is estimated at 1:1000–1:3000 in Ashkenazi Jews [32] and less than 1:30,000 in non-Jews [33], inclusion of additional Jewish subjects may have increased our percentage of ΔGAG cases.

Our study was limited to interrogation of Exon 5 of *TOR1A *and does not exclude a role for *TOR1A *mutations and/or variants in the etiopathogenesis of late-onset primary dystonia. Single nucleotide polymorphisms (SNPs) within or in close proximity to the 3' UTR of *TOR1A *may be associated with primary focal dystonia [34-36]. Mutations in Exons 1–4 and associations between dystonia phenotypes and *TOR1A *copy number variants and single nucleotide polymorphisms must be interrogated in future studies.

Previous studies of primary dystonia have employed a wide variety of methods for detection of *TOR1A *mutations [13,21,37-49]. For large cohorts, direct sequencing of each sample is expensive, labor intensive, and inefficient. Similarly, previously employed techniques such as PCR-based polyacrylamide gel electrophoresis (PAGE) with silver staining [37], restriction fragment length polymorphism (RFLP) analysis with restriction enzyme BseR1 [38-48] and SSCP (single-strand conformation polymorphism) analysis [13,21], are slow, requiring multiple steps including gel electrophoresis. Although not gel-dependent, denaturing high-performance liquid chromatography (DHPLC) also requires post-PCR handling which is labor-intensive and associated with the risk of well-to-well cross contamination [49].

HRM has recently been introduced as a screening method for mutation detection. HRM is a closed-tube method that can be performed in a rapid, economical, reliable, and high-throughput fashion. In a comparative study, Chou and colleagues showed that HRM is superior to DHPLC [50]. HRM has been utilized for the detection of germline and somatic mutations as well as SNP genotyping with high sensitivity and specificity [23,51-54]. Other HRM applications include microsatellite analysis, screening for loss of heterozygosity, and DNA methylation analysis.

The utility and efficiency of HRM can be maximized by careful experimental design. In our study, a single pair of primers was designed to cover the entire coding region of *TOR1A *Exon 5 and generated a 314 bp amplicon. Theoretically, differentiation of samples carrying a sequence variation is better with shorter amplicons. In general, amplicons from 50 to 250 bp are ideal for HRM, particularly when employed for SNP and homozygous mutation analysis. However, 100% sensitivity and specificity has been observed with amplicons larger than 600 bp [55]. Thus, the clear separation of 314 bp amplicons harboring heterozygous ΔGAG and c.863G>A mutations was not unexpected.

HRM offers additional advantages over older screening techniques. First, amplicons of interest only require purification prior to follow-up sequencing. There is no need to PCR amplify DNA templates. Second, HRM robustly differentiates genotypes despite moderate differences in well-to-well concentrations of DNA templates. Lastly, Roche's LightCycler^® ^480 Real-Time PCR system offers 384-well plate capabilities which, when coupled with a robotic workstation, permits completion of very large projects (>10,000 samples) in a single week. Clearly, HRM is well-suited for many candidate gene and SNP-association studies of dystonia and other neurological disorders.

## Conclusion

In conclusion, we have developed a fast, efficient and reliable screening method for known mutations (ΔGAG and c.863G>A) within the coding region of *TOR1A *Exon 5 using HRM. HRM showed 100% diagnostic sensitivity and specificity. In a large cohort of patients with non-generalized primary dystonia in the US, *TOR1A *Exon 5 mutations were very uncommon; only two classic DYT1 ΔGAG and no c.863G>A mutations were identified. HRM may be applicable to high-throughput mutation detection in other movement and general neurological disorders.

## Competing interests

The authors declare that they have no competing interests.

## Authors' contributions

MSL designed the study. JX, SG and YZ extracted DNA from blood samples. JX and MSL optimized and implemented the HRM protocol and analyzed HRM data. RWB, JSP, BAR, SDT, MK, RP, AB, ZKW, RJU, PH, DKS, DT, DDT, KPF, RFP and MSL examined research subjects. SDB provided DNA from subjects with a clinical diagnosis of dystonia. JX and MSL drafted the manuscript. All authors read and approved the final manuscript.

## Pre-publication history

The pre-publication history for this paper can be accessed here:


